# Large multicenter randomized trials in autism: key insights gained from the balovaptan clinical development program

**DOI:** 10.1186/s13229-022-00505-6

**Published:** 2022-06-11

**Authors:** Suma Jacob, Evdokia Anagnostou, Eric Hollander, Roger Jou, Nora McNamara, Linmarie Sikich, Russell Tobe, Declan Murphy, James McCracken, Elizabeth Ashford, Christopher Chatham, Susanne Clinch, Janice Smith, Kevin Sanders, Lorraine Murtagh, Jana Noeldeke, Jeremy Veenstra-VanderWeele

**Affiliations:** 1 Department of Psychiatry and Behavioral Sciences, University of Minnesota, Minneapolis, MN USA; 2 Holland Bloorview Kids Rehabilitation Hospital, University of Toronto, Toronto, ON Canada; 3 Department of Psychiatry and Behavioral Sciences, Albert Einstein College of Medicine, New York, NY USA; 4 Child Study Center, Yale School of Medicine, New Haven, CT USA; 5 Department of Psychiatry, University Hospitals, Cleveland, OH USA; 6 Department of Psychiatry and Behavioral Sciences, Duke Clinical Research Institute, Duke University School of Medicine, Durham, NC USA; 7 Nathan Kline Institute for Psychiatric Research, Orangeburg, NY USA; 8 Kings College London, London, UK; 9 David Geffen School of Medicine at UCLA, Los Angeles, CA USA; 10 F. Hoffmann-La Roche Ltd, Welwyn Garden City, UK; 11 F. Hoffmann-La Roche Ltd, Basel, Switzerland; 12 F. Hoffmann-La Roche Ltd, Genentech, South San Francisco, CA USA; 13 Columbia University and New York State Psychiatric Institute, New York, NY USA

**Keywords:** aV1ation, V1aduct, VANILLA, Autism spectrum disorder, Placebo response, Balovaptan

## Abstract

**Background:**

Autism spectrum disorder (ASD) is a common and heterogeneous neurodevelopmental condition that is characterized by the core symptoms of social communication difficulties and restricted and repetitive behaviors. At present, there is an unmet medical need for therapies to ameliorate these core symptoms in order to improve quality of life of autistic individuals. However, several challenges are currently faced by the ASD community relating to the development of pharmacotherapies, namely in the conduct of clinical trials. Balovaptan is a V1a receptor antagonist that has been investigated to improve social communication difficulties in individuals with ASD. In this viewpoint, we draw upon our recent first-hand experiences of the balovaptan clinical development program to describe current challenges of ASD trials.

**Discussion points:**

The balovaptan trials were conducted in a wide age range of individuals with ASD with the added complexities associated with international trials. When summarizing all three randomized trials of balovaptan, a placebo response was observed across several outcome measures. Placebo response was predicted by greater baseline symptom severity, online recruitment of participants, and less experienced or non-academic trial sites. We also highlight challenges relating to selection of outcome measures in ASD, the impact of baseline characteristics, and the role of expectation bias in influencing trial results.

**Conclusion:**

Taken together, the balovaptan clinical development program has advanced our understanding of the key challenges facing ASD treatment research. The insights gained can be used to inform and improve the design of future clinical trials with the collective aim of developing efficacious therapies to support individuals with ASD.

**Supplementary Information:**

The online version contains supplementary material available at 10.1186/s13229-022-00505-6.

## Background

Autism spectrum disorder (ASD) is an etiologically and clinically heterogeneous neurodevelopmental condition estimated to affect ~ 2% of the US population [1, 2]. It is characterized by the core symptoms of social communication difficulties and repetitive and restricted behaviors, which frequently impact quality of life [2, 3]. There are several associated symptoms and co-occurring conditions including intellectual disability, anxiety, attention deficit hyperactivity disorder (ADHD), depression, unusual immune functioning, and gastrointestinal dysfunction [2].

There are currently no evidence-based pharmacologic therapies to ameliorate core ASD symptoms. Current therapies for ASD include a limited number of behavioral interventions primarily targeted toward the individual needs of young autistic children [4]. Studies of these approaches focus on outcomes not directly related to core reciprocal socialization difficulties, such as language, cognition, adaptive behaviors, or associated symptoms [4–6]. Furthermore, there are often waiting lists to access behavioral therapists, difficulties in accessing funding, and challenges balancing the intensive time demands of therapy with other household responsibilities [5, 7]. Only aripiprazole and risperidone have been approved by the US Food and Drug Administration (FDA) and are indicated in treatment of irritability and agitation associated with pediatric ASD, rather than for core symptoms [2].

**Fig. 1:**
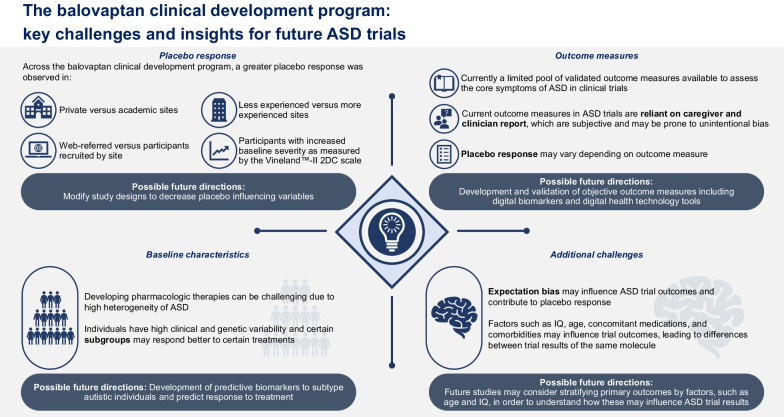
The balovaptan clinical development program: key challenges and insights for future ASD trials. *2DC* two-domain composite, *ASD* autism spectrum disorder, *IQ* intelligence quotient

There is a need for pharmacologic therapies to supplement current approaches to target the core symptoms of ASD. However, there are several challenges to the conduct of robust clinical trials that assess the efficacy of novel pharmacologic interventions (Fig. 1). Here, we discuss and examine key challenges including placebo response, impact of baseline characteristics, and selection of appropriate outcome measures in the context of the largest clinical trials program in ASD to date.

## The balovaptan clinical development program

The balovaptan clinical development program was planned in collaboration with the ASD community (advocates, caregivers, and autistic individuals), to assess the effect of balovaptan, a vasopressin 1a (V1a) receptor antagonist, on reducing social communication difficulties in autistic individuals. The vasopressin system has previously been implicated in social behavior in humans and rodents [8–10], and upon identification and administration of the V1a receptor antagonist RG7713, autistic adults showed improvements in social communication as measured by eye tracking and emotion identification [11, 12]. Hence, there was strong rationale for investigating balovaptan for ASD.

Vasopressin ANtagonist to Improve SociaL Communication in Autism (VANILLA [NCT01793441]) was a Phase 2, 12-week, double-blind, placebo-controlled trial to assess the safety, tolerability, and efficacy of balovaptan 1.5 mg (*n* = 32), 4 mg (*n* = 77), and 10 mg (*n* = 39) in 223 autistic men (18–45 years). Mean (standard deviation [SD]) age was 24.7 (6.3) years in the placebo group and 28.2 (7.8), 24.5 (6.6), and 23.9 (5.0) in the balovaptan 1.5, 4, and 10 mg groups, while mean intelligence quotient (IQ) was 96.6 (15.1) in the placebo group, and 100.1 (17.5), 99.5 (17.2), and 97.3 (17.8) in the balovaptan 1.5, 4, and 10 mg groups, respectively [8]. The Social Responsiveness Scale, 2nd Edition (SRS-2) primary endpoint showed improvements across all arms including placebo, although no significant differences were observed in mean change from baseline between arms. However, improvements for balovaptan 4 mg and 10 mg versus placebo (*n* = 75) were observed on the Vineland™-II Adaptive Behavior Composite score, which were largely driven by the Socialization and Communication domains of Vineland™-II. A post hoc analysis of VANILLA participants using a composite measure of the Vineland™-II Socialization and Communication domains (two-domain composite [2DC]) showed significant improvements with balovaptan 10 mg versus placebo. The only other improvement observed across all endpoints assessed was for balovaptan 10 mg versus placebo on the Pediatric Quality of Life™ (PedsQL™) Generic Core Scale exploratory endpoint [8].

V1aduct (Phase 3; NCT03504917) and aV1ation (Phase 2; NCT02901431) were 24-week, randomized, double-blind, placebo-controlled trials that assessed the efficacy and safety of balovaptan 10 mg in 321 autistic adults (aged ≥ 18 years) and the equivalent dose (i.e., an age-adjusted dose of balovaptan providing exposure approximately equal to a 10 mg adult dose) in 167 autistic pediatric participants (primary analysis population; aged 5–17 years), respectively, compared with placebo [13, 14]. In V1aduct, mean (SD) age was 27.6 (9.8) years in the placebo group and 27.6 (9.7) in the balovaptan group, while mean IQ was 106.1 (18.5) in the placebo group and 103.6 (17.6) in the balovaptan group, respectively [13]. In aV1ation, mean age (SD) was 12.3 (3.4) in the placebo group and 11.9 (3.5) in the balovaptan group [14]. Although both balovaptan and placebo treatments resulted in improvements in the primary endpoint, the Vineland™-II 2DC score at week 24, in V1aduct (balovaptan *n* = 163 treated; placebo *n* = 158) and aV1ation (balovaptan *n* = 86; placebo *n* = 81), there were no significant differences between the balovaptan 10 mg and placebo groups. Similarly, no differences between balovaptan 10 mg or 10 mg equivalent versus placebo were seen in any of the secondary endpoints. V1aduct was terminated early, due to futility analysis after 50% of individuals completed the week 24 visit (*n* = 181). Balovaptan was well tolerated, and no safety concerns were identified across all three trials. An additional file shows all primary and secondary efficacy objectives for VANILLA, V1aduct, and aV1ation (See Additional file 1). Full baseline characteristics and endpoint data are available in the VANILLA, V1aduct, and aV1ation publications [8, 13, 14]. To note, Autism Diagnostic Observation Scale (ADOS-2) was included as a baseline characteristic and was used to confirm diagnosis in aV1ation and V1aduct [8, 13, 14].

In summary, the balovaptan clinical development program did not find that balovaptan was efficacious in improving social communication difficulties. While the reasons for lack of balovaptan efficacy are unknown, we hypothesize that several factors, such as placebo response, may have contributed to the observed lack of treatment effect. Factors that may have influenced the results will be described further in subsequent sections of this article.

## What is the role of the placebo response in ASD trials?

A placebo effect is defined as a change in underlying neurobiologic and psychologic mechanisms of expectancies following administration of an inactive treatment, while a placebo response is defined as a change in an individual’s condition and/or symptoms [15, 16]. Several factors have been hypothesized to increase placebo response, e.g., rater bias, which can be characterized by inflation of baseline scores and a tendency to observe improvement over time [17, 18]. Placebo response has been observed across multiple randomized controlled trials (RCTs) of pharmacologic and dietary agents in autistic children and adolescents [19]. Placebo response has been identified as an ongoing challenge in a wide variety of neuropsychiatric conditions, including major depressive disorder [20, 21], mood and anxiety conditions [22], schizophrenia [23, 24], and Fragile X Syndrome [25].

Several strategies have previously been implemented to reduce placebo response, with limited success. For example, placebo lead-in phases have been utilized with the intention of identifying and excluding participants who demonstrated response to placebo [19]. While this methodology has potential to reduce placebo response, literature to date suggests that this approach may not be beneficial, and recent meta-analyses in ASD have noted that there is not yet sufficient data to examine the benefits of a placebo lead-in phase [19, 23].

A placebo response was observed across various outcome measures in all three balovaptan trials, despite placebo management video training and education delivered in-person to sites, investigators, participants, and caregivers prior to and during aV1ation and V1aduct (this training was not delivered for VANILLA). To note, training for investigators was delivered via training videos, modules, and at investigator meetings. The training addressed investigator–participant interactions, how to manage informed consent, and the potential impacts of social media posting. Placebo management training was conducted via an external agency and aimed to be state-of-the-art for ASD and general psychiatry practices.

### Impact of site experience with balovaptan

All sites involved in the balovaptan clinical development program had experience previously conducting at least one ASD clinical trial. In V1aduct, those recruited to sites with no specific balovaptan trial experience (*n* = 92 participants, 18 sites) had a greater placebo response than those recruited to the more experienced sites, i.e., those who had received prior training in at least one other balovaptan trial (*n* = 89 participants, 12 sites). In the futility analysis population at week 24, mean change from baseline in Vineland™-II 2DC scores with balovaptan versus placebo was 5.65 (8.60) versus 5.07 (9.85), respectively, for experienced sites compared with 3.38 (11.39) versus 7.43 (14.21), respectively, for less experienced sites.

While centralized training was delivered to all raters and bespoke refresher training was delivered to poor performing raters of Vineland™-II 2DC, these results suggest that site experience should be considered when designing clinical trials and additional training may be required for raters at less experienced sites. Poor performing raters were identified through evaluation of scoring on Vineland™-II assessment and corresponding audio recordings. Evaluation was carried out by individuals independent of the site and sponsor. These individuals were specialized in the Vineland and cross-calibrated with each other. Only sites with ASD experience were selected for VANILLA, yet a large placebo response was observed on the SRS-2 and as such, we cannot discount that other factors may also influence outcomes. To note, specific standards for experience and education in utilized scales were predefined for accepting raters onto the V1aduct and aV1ation studies. Inter-rater reliability was benchmarked against a standard and assessed specifically via the Vineland™-II Scale.

Future approaches to improve rater reliability include the use of blinded centralized raters who can interview participants at baseline and throughout the study through teleconferencing and/or video conferencing approaches. Blinded centralized raters may also reduce rater bias and reduce overall placebo response compared with raters based at study sites [17, 18].

### Impact of site type

A greater placebo response was observed for individuals recruited to private (specialist clinical research sites whose funding was predominantly or entirely provided by performing clinical trials for sponsors/pharmaceutical companies) compared with academic (specialist research centers supported by government, academia, and industry where sponsored funding and treatment were provided) research centers in both V1aduct and aV1ation at week 24. In V1aduct, change from baseline (SD) in Vineland™-II 2DC score at week 24 in private versus academic research sites, respectively, was 4.6 (9.8) versus 3.2 (13.0) with balovaptan compared with 7.4 (13.1) versus 2.5 (7.9) with placebo. V1aduct had substantially fewer individuals recruited to academic (*n* = 34, 14 sites) versus private (*n* = 147, 16 sites) sites. Private sites were activated earlier partly due to use of centralized institutional review boards (IRBs) and rapid contract establishment pathways. Notably, in VANILLA, there were 154 participants at 19 academic sites versus 69 participants at seven private sites.

In aV1ation, 65 individuals were randomized at 20 private sites, while 67 were randomized at 21 academic sites. Change from baseline in Vineland™-II 2DC scores was similar between private and academic sites (2.1 [9.5] vs. 2.1 [7.6], respectively) in the balovaptan arm, whereas there was a greater numerical change at private versus academic sites (4.2 [9.0] vs. 2.9 [7.0]) in the placebo arm.

Academic sites may be more likely to have expert knowledge of the Vineland™-II 2DC Scale (through prior involvement in a Roche ASD trial), be familiar with participants and families, and have more overall experience in the assessment of ASD. This may be due to a lower turnover rate of investigators, more stringent training procedures, and subsequently more experienced staff [25]. Meta-analyses of several neuropsychiatric trials assessing various pharmacotherapies for major depressive disorder, schizophrenia, and anxiety have similarly identified that a higher proportion of participants recruited from academic sites and academic-funded trials predict a lower rate of placebo response [22, 26, 27]. Moreover, additional site factors, such as a larger number of study sites and fewer participants per site, have been positively correlated with increased placebo response in other neuropsychiatric conditions [22, 28].

We propose several ways to further engage with and optimize participation of academic sites, including: (1) leveraging contacts with academic centers of excellence and implementing faster contracting with academic sites; (2) creating effort payment structures and recruitment milestones that parallel grant budgets for non-industry clinical research; (3) identifying obstacles to participation in trials sponsored by pharmaceutical companies; and (4) use of centralized IRBs, where possible. Participants enrolled at private sites may experience a greater novelty and expectation bias due to less prior interaction with healthcare systems and research studies, which may impact upon their ratings. Importantly, no clear differences in participant baseline characteristics between site types were observed. The IQ of individuals recruited to private sites was numerically higher than individuals recruited to academic sites; however, the overlap in SD between private and academic sites means no conclusion can be made about this factor.

### Impact of method of referral

In V1aduct, a greater change from baseline in Vineland™-II 2DC score was observed in participants who were recruited via study-specific web referrals. Web-based screening forms for potential participants were collected via an independent vendor and if appropriate for inclusion, details were provided to sites. ASD diagnoses were confirmed with the ADOS-2 by a certified rater. At week 24, for participants known by sites (*n* = 63) versus those referred via web (*n* = 71, includes four participants recruited via advocacy and healthcare professional referrals), mean (SD) change from baseline on the Vineland™-II 2DC Scale in the balovaptan arm was 4.1 (10.6) versus 4.4 (11.4) compared with 4.5 (11.3) versus 10.9 (15.3) in the placebo arm. For those recruited by web referral, the rater may have lacked rapport and knowledge of individual developmental and longitudinal histories in identifying and rating changes, relative to established patients known to the site. Those seeking study-specific web referrals may have been more proactive in researching trials, leading to greater enthusiasm and expectation for a positive trial result. Interestingly, only private sites recruited participants via the web, indicating that academic sites may utilize established recruitment pools of individuals, potentially contributing to the lower placebo response observed in academic compared with private sites. Similar proportions of web-referred participants were recruited to experienced private sites (*n* = 36 participants) versus less experienced private sites (*n* = 31 participants) in V1aduct. These findings indicate that it may be beneficial for future trials to reduce the number of web-referred participants in parallel with recruiting a higher proportion of individuals who are known by sites. However, the strengths of web referrals should be considered, such as the potential for improved time efficiency and cost-effectiveness compared with offline recruitment [29].

Differences in response between site types/experiences/referral methods tended to be most evident in the placebo arm rather than the balovaptan arm. While we were unable to identify a reason for this, a previous ASD study investigating the efficacy of citalopram in autistic children found that the placebo response was largely driven by those with less severe versus more severe symptoms [30].

## How can baseline factors influence ASD trial results?

### Heterogeneity of autistic individuals poses a significant challenge

Development of pharmacologic therapies can be challenging due to the heterogeneity of ASD pathophysiology [2]. Heterogeneity in genotypes is a significant challenge, especially since ~ 25% of autistic individuals have a rare genetic variant of major effect, but no single mutations are present in > 1% of the ASD population. Common genetic variants with small effects are thought to have additive effects, leading to the development of complex ASD traits and further heterogeneity [31].

One consideration for the balovaptan clinical development program is whether participants’ response to balovaptan may have varied depending on underlying heterogeneity, such as common genetic polymorphisms in *AVPR1A*, the gene encoding the V1a receptor. While current data are limited, some studies have suggested an association between polymorphisms in or near the *AVPR1A* gene region and ASD, although this has not reached statistical significance at the genome-wide level [32]. Future directions include working toward identifying genetic subtypes of ASD that are relevant to the biology of the pharmaceutical agent being tested. This will enable targeting of therapies to groups of individuals who may experience the most benefit. Several studies are underway to identify biomarkers predictive of treatment response [33]. Furthermore, it is plausible that while balovaptan alone may not improve social communication, social skills training augmented by balovaptan treatment may lead to better outcomes. This was suggested in a recent article discussing the potential benefits of oxytocin treatment in parallel with behavioral interventions for ASD [34].

### Lower baseline adaptive skills may be associated with greater placebo response

Across all trials, Vineland™-II 2DC scores < 60 (i.e., lower baseline adaptive skills) compared with scores ≥ 60 were associated with greater improvements in socialization and communication in both the balovaptan and placebo arms following treatment, as measured by the Vineland™-II 2DC Scale.

In VANILLA, change from baseline (SD) to week 12 in Vineland™-II 2DC with balovaptan 10 mg versus placebo was 5.1 (5.8) versus 2.0 (7.9) for participants with baseline Vineland™-II 2DC ≥ 60 and 15.3 (22.0) versus 2.8 (6.4), respectively, for those with baseline Vineland™-II 2DC < 60 (balovaptan baseline Vineland™-II 2DC score range 28–86; placebo 20–96). Across the balovaptan arm, a weak correlation between baseline Vineland™-II 2DC score and mean change from baseline Vineland™-II 2DC was found (Pearson’s correlation coefficient [*r*] =  − 0.18) in VANILLA. However, for V1aduct and aV1ation, moderate correlations were observed (V1aduct scores at weeks 12 and 24, respectively, were *r* =  − 0.44 and − 0.41, and *r* =  − 0.40 and − 0.31 for aV1ation). In V1aduct, for those with baseline Vineland™-II 2DC ≥ 60, mean change from baseline (SD) in Vineland™-II 2DC for balovaptan versus placebo was 3.3 (10.5) versus 3.2 (9.7), respectively, whereas participants with baseline Vineland™-II 2DC < 60 had scores of 7.2 (9.4) versus 17.0 (14.3), respectively (balovaptan baseline Vineland™-II 2DC score range 32–100; placebo 20–106). It is possible that those with a higher baseline Vineland™-II 2DC score had less scope for improvement due to the limited number of questions relating to higher levels of adaptive functioning. The range for improvement on Vineland™-II 2DC varies across age groups, which may have also influenced outcomes. While a correlation between Vineland™-II 2DC scores and IQ has not been determined, recruiting individuals with IQ < 70 in future trials (all three trials included participants with IQ ≥ 70) or comorbid intellectual disability may allow more range for improvement on the Vineland™-II 2DC Scale.

## What are the challenges related to outcome measures in ASD clinical trials?

### Variation in trial design may influence outcome measure placebo response

While the Vineland™-II 2DC Scale was subject to a marked placebo response in both aV1ation and V1aduct, no such placebo response was observed on the Vineland™-II Aberrant Behavior Checklist when utilized as a secondary endpoint in VANILLA for balovaptan 4 mg and 10 mg doses [8, 13, 14]. Coupled with the substantial placebo response observed on the SRS-2 primary endpoint of VANILLA, these data indicate that primary endpoints are prone to placebo response. Several secondary endpoints also showed placebo response across the balovaptan trials, including the Aberrant Behavior Checklist—Lethargy/Social Withdrawal (ABC-L/SW) Subscale, the Hamilton Anxiety Rating Scale, the patient-reported PedsQL™ Generic Core Scale, and the Clinical Global Impression—Improvement (CGI-I) and Clinical Global Impression—Severity Scales [8, 13, 14]. Of note, change in raters could have influenced overall Vineland™-II 2DC results in aV1ation and V1aduct (V1aduct overall rater change, *n* = 33 [10.3%]; aV1ation overall rater change, *n* = 35 [17.9%]).

The extent of the placebo response for different outcome measures varied between trials. For example, a substantial placebo response was observed in aV1ation and V1aduct on the CGI-I; however, in VANILLA, a less marked placebo response on the CGI-I was observed [8, 13, 14].

Across the three balovaptan trials, clinician- and caregiver-reported outcomes appeared to be equally affected by placebo response. However, a recent meta-analysis of 86 ASD RCTs identified that caregiver ratings were associated with a greater placebo response compared with clinician ratings [19]. This could be due to a placebo-by-proxy effect, whereby the caregiver’s knowledge that the autistic individual may be receiving treatment alters perception of symptoms or behavior in the autistic individual [19]. Particularly for Vineland™-II, individual questions could make caregivers more sensitive to behaviors that may not typically be noted during previous visits, leading to inflation of Vineland™-II scores as the trial progresses. For example, on the first administration of the Vineland™-II, caregivers might be uncertain whether a participant can follow three-part instructions, leading them to check this before the next administration. A smaller meta-analysis of 26 pediatric ASD pharmacologic and dietary supplement RCTs, however, reported the opposite: clinician-rated measures were more likely to be subject to a placebo response, which the authors attributed in part to enthusiasm or motivation for positive results [35].

### Selection of appropriate outcome measures to assess the core symptoms of ASD is challenging

There are few widely accepted and validated outcome measures available to evaluate socialization and communication in autistic individuals, and often there is limited knowledge on how reliable and sensitive they are for detecting change [36, 37].

In 2013, a review found that a total of 253 outcome measures of cognitive/behavioral symptoms had been used across 195 ASD trials between 2001 and 2010. Remarkably, 61.6% of these outcome measures were used in only one trial [38]. The scales most commonly utilized as outcome measures, used in 3.9–5.0% of the 195 trials assessed, were the Aberrant Behavior Checklist, Vineland™-II, and CGI scales [38]. The lack of validated outcome measures and lack of consensus on which are most appropriate may in part be due to the limited number of previous studies on pharmacologic therapies to treat the core symptoms of ASD.

An expert panel previously supported only six outcome measures as appropriate for use in pediatric ASD trials for measuring social communication [36]. Those deemed appropriate for assessing such concepts of interest included the ABC-L/SW Subscale and the Vineland™-II Socialization Scale [36]. To note, ABC-L/SW was not a primary outcome measure in the balovaptan studies. It was suggested that the ABC-L/SW Subscale had the strongest empirical support as an outcome measure to assess socialization. Despite this, the Vineland™-II 2DC Scale appeared to outperform the ABC-L/SW Subscale in terms of placebo response in aV1ation [14]. The empirical support for the ABC-L/SW may be weighted toward its use in risperidone and aripiprazole trials [39, 40], where improvements in irritability and agitation may have enabled more successful social interaction without necessarily improving core symptoms.

### Future approaches to ASD outcome measures

Current outcome measures in ASD trials are primarily reliant on informant report. While this can provide a rich source of information, interpretation can be subjective, prone to unintentional bias, and may vary across raters [41].

There is significant interest in defining objective measures of social behavior or cognition, as well as valid, reliable biomarkers to assess clinically relevant change in the core symptoms of ASD [42, 43]. Objective measures of behavior include eye tracking and machine learning applied to video recordings of non-verbal communication or social interactions [44]. Neurocognitive testing could also reveal reliable changes in social information processing or cognition; however, it is important to evaluate the frequency of testing required and sensitivity to change of these measures [45]. Additional biomarkers include electroencephalography and functional magnetic resonance imaging [43, 46]. These approaches require further research to assess their translatability as indicators of clinically relevant change, and do not necessarily index change in real-world function, which may support FDA or European Medicines Agency approval [47, 48]. Several initiatives, including those led by the European Autism Interventions-A Multicentre Study for Developing New Medications (EU-AIMS) and the Autism Biomarkers Consortium for Clinical Trials (ABC-CT), aim to characterize and validate biomarkers for use in ASD trials [33, 46]. Other ongoing studies, including oRBiting (NCT03611075), aim to characterize biomarkers and outcome measures primarily for the assessment of restricted and repetitive behaviors in addition to social communication and interaction [49]. The development of digital health technology tools has a strong potential to integrate into clinical research and may be a robust and sensitive means to measure efficacy of pharmacologic interventions [50]. Digital health technology tools may also enable the assessment of ASD characteristics in everyday settings to capture clinically meaningful change. Based upon the Autism and Beyond and iOS ResearchKit studies, a digital app was developed for caregivers to collect videos of their children while watching a movie. The videos can be uploaded and then analyzed to quantify children’s behaviors and emotions [51]. The Janssen Autism Knowledge Engine (JAKE^®^) has been developed to measure the core and associated symptoms of ASD, comprising a mobile app and wearable sensors to track progress, core symptoms, and physiologic characteristics [52]. Roche has started to develop a suite of assessments collected via consumer smartphones and wearables to allow objective and daily assessment of ASD core symptoms and potential underlying adaptive and cognitive skills, with an aim of using these assessments to monitor ASD symptoms in RCTs [53]. Digital health technology tools are also being explored as a means to support social communication in autistic individuals [54]. Other avenues that could be explored include novel outcome measures such as the Brief Observation of Social Communication Change, which was developed for young autistic children and aims to quantify subtle changes in social communication [55]. Caregiver- and participant-reported exit interviews may also be a valuable way to obtain both qualitative and quantitative data, which may support the development of novel measurement strategies, such as biomarkers, to evaluate meaningful change in ASD outcomes.

## Additional insights from the balovaptan clinical development program

### Expectation bias may influence ASD trial outcomes

An individual’s expectation of improvement may influence trial outcomes, whether considering autistic individuals, caregivers, or clinicians, thereby driving placebo response. Expectation bias in the balovaptan clinical development program may have been present due to various factors: (1) balovaptan was one of the first medications in clinical development to target the core symptoms of ASD, causing excitement within the ASD community; (2) high expectations of balovaptan may have been held by participants, caregivers, and clinicians due to the improvements reported in VANILLA; and (3) the FDA breakthrough designation of balovaptan following VANILLA may have influenced the aV1ation and V1aduct placebo response. Expectation bias has also been shown to mediate placebo response in antidepressant clinical trials [56]. Strategies to manage and assess participant expectations may be implemented in trials moving forwards [56, 57]; a potential informative way to predict expectation bias could be the use of participant/caregiver questionnaires.

### Several factors may have led to differences in balovaptan trial outcomes

There were clear differences between outcomes in the three balovaptan trials, which may be due to differences in trial population baseline characteristics. For example, VANILLA recruited only autistic men aged 18–45 years (mean [SD] age of 23.9 [5.0] and 24.7 [6.3] years in balovaptan 10 mg and placebo arms, respectively), V1aduct recruited both men and women with no upper age limit (mean [SD] age of 27.6 [9.7] years), and aV1ation recruited children and adolescents aged 6–17 years (mean [SD] age of 12.6 [2.9]) [8, 13, 14]. Concomitant medications, IQ, and the number of individuals with at least one known comorbidity were similar across all three trials, with the exception of a larger proportion of individuals with ADHD and taking stimulants in aV1ation compared with V1aduct and VANILLA. A large proportion of the aV1ation study population had a comorbid diagnosis of ADHD (68.8% receiving placebo, 61.3% receiving balovaptan), of which the majority were taking psychostimulants (65.2% receiving placebo, 69.4% receiving balovaptan). Individuals and families with previous positive experiences with medications may have had an increased expectation bias for a trial of a new medication.

Additionally, study intensity and duration varied between trials, whereby participants in aV1ation and V1aduct were subject to less frequent visits but a longer treatment duration compared with VANILLA. Future studies may consider stratification of primary outcomes by age, sex, and IQ to better understand how these may influence treatment response. It is important, however, to note that variability between ASD studies is likely to be a constant challenge given the substantial heterogeneity across the ASD population.

## Conclusions

While the totality of data across all three trials indicates that balovaptan does not show efficacy in improving social communication in the populations assessed, these trials span a broad age range and are among the largest biomedical RCTs in ASD to date. Our observations highlight some drivers of high placebo response in ASD trials, while demonstrating the need for robust objective outcome measures that are sensitive to change. Enrollment criteria that limit ASD heterogeneity may increase the likelihood of detecting a treatment response.

While the search for appropriate outcome measures continues, interim solutions on how to plan and conduct trials are key to advancing the field and improving overall care for autistic individuals [58]. This may be achieved through various avenues such as harnessing stakeholder expertise in clinical trial outcome and design and improving the ability to identify treatment response mediators [58]. Additionally, the use of Sequential Multiple Assignment Randomized Trials can enable researchers to carry out multiple randomizations and evaluate adaptive interventions, while providing detailed data on optimal treatment regimens on a participant-by-participant basis [59, 60].

Gaining a better understanding of optimal trial design in a broad ASD clinical population will be integral for future ASD trials, and the development of novel drugs shown to benefit core symptoms will further contribute to our overall understanding. These findings could be important for other neuropsychiatric disorder clinical trials, whereby factors influencing placebo response and challenges relating to outcome measures may be applicable and generalizable to different populations of individuals.

## Supplementary Information

**Additional file 1. Table S1**. Primary and secondary efficacy objectives in the VANILLA, aV1ation, and V1aduct clinical trials.

## Data Availability

For up-to-date details on Roche's Global Policy on the Sharing of Clinical Information and how to request access to related clinical study documents, see here: https://go.roche.com/data_sharing. Request for rater change data, and data stratified by site experience, site type, referral method, and baseline adaptive skills underlying this publication requires a detailed, hypothesis-driven statistical analysis plan that is collaboratively developed by the requestor and company subject matter experts. Such requests should be directed to datarequest.autism@roche.com for consideration. Anonymized records for individual patients across more than one data source external to Roche cannot, and should not, be linked due to a potential increase in risk of patient re-identification.

## Abbreviations

2DC: Two-domain composite
ABC-CT: Autism Biomarkers Consortium for Clinical Trials
ABC-L/SW: Aberrant Behavior Checklist—Lethargy/Social Withdrawal
ADHD: Attention deficit hyperactivity disorder
ASD: Autism spectrum disorder
CGI-I: Clinical Global Impression—Improvement
EU-AIMS: European Autism Interventions-A Multicentre Study for Developing New Medications
FDA: Food and Drug Administration
IQ: Intelligence quotient
IRB: Institutional review board
JAKE^®^: Janssen Autism Knowledge Engine
PedsQL™: Pediatric Quality of Life™
RCT: Randomized controlled trail
SD: Standard deviation
SRS-2: Social Responsiveness Scale, 2nd Edition
V1a: Vasopressin 1a
VANILLA: Vasopressin ANtagonist to Improve SociaL Communication in Autism
