# Supplementary material for: Large multicenter randomized trials in autism: key insights gained from the balovaptan clinical development program
Source: Mol Autism. 2022 Jun 11;13:25. doi: 10.1186/s13229-022-00505-6 (PMC9188723; doi:10.1186/s13229-022-00505-6)
Supplement: Supplementary file 1 — Additional file 1. Table S1. Primary and secondary efficacy objectives in the VANILLA, aV1ation, and V1aduct clinical trials. [file 13229_2022_505_MOESM1_ESM.pdf]

Supplement to: Large multicenter randomized trials in autism: key insights gained from the balovaptan clinical development program

Suma Jacob, Evdokia Anagnostou, Eric Hollander, Roger Jou, Nora McNamara, Linmarie Sikich, Russell Tobe, Declan Murphy, James McCracken, Elizabeth Ashford, Christopher Chatham, Susanne Clinch, Janice Smith, Kevin Sanders, Lorraine Murtagh, Jana Noeldeke, and Jeremy Veenstra-VanderWeele.

Table S1. Primary and secondary efficacy objectives in the VANILLA, aV1ation, and V1aduct clinical trials.

Efficacy objective	Endpoint
**VANILLA**	
Primary efficacy objective	• Change from baseline at week 12 on the SRS-2
Secondary efficacy objective	• Change from baseline at week 12 in the Vineland™-II<br>• Change from baseline at week 12 in the Vineland™-II, ABC, RBS-R, ADAMS, and STAI<br>• Change from baseline at week 12 in the CGI-I and CGI-S<br>• Clinical response (CGI-S score of 1 or 2*)
**aV1ation**	
Primary efficacy objective	• Change from baseline at week 24 on the Vineland™-II 2DC score
Secondary efficacy objective	• Change from baseline at weeks 12 and 24 on the Vineland™-II composite standard score<br>• Change from baseline at weeks 12 and 24 in the Vineland™-II Communication, Socialization, and Daily Living Skills domains<br>• Proportion of participants with a ≥6-point improvement in Vineland™-II 2DC score<br>• Change from baseline at weeks 12 and 24 in the CGI-S and OACIS-S<br>• Improvements in clinical impressions at weeks 12 and 24 as measured by the CGI-I and OACIS-I<br>• Change from baseline at weeks 12 and 24 in the PedsQL™ Generic Core Scale<br>• Change from baseline at weeks 12 and 24 in the Vineland™-II composite standard score in adults and children independently<br>• Change from baseline at week 12 on the Vineland™-II 2DC
**V1aduct**	
Primary efficacy objective	• Change from baseline at week 24 on the Vineland™-II 2DC score
Secondary efficacy objective	• Change from baseline at week 12 on the Vineland™-II 2DC score<br>• Change from baseline at weeks 12 and 24 in the Vineland™-II ABC standard score<br>• Change from baseline at weeks 12 and 24 in the Vineland™-II Socialization, Communication, and Daily Living Skills domains<br>• Change from baseline at weeks 12 and 24 in the PedsQL™ Core module summary and total scores<br>• Change from baseline in CGI-S at weeks 12 and 24<br>• Improvements in CGI-I at weeks 12 and 24<br>• Change from baseline in the HAM-A total and domain scores at weeks 12 and 24<br>• Proportion of participants with a ≥6-point improvement in Vineland™-II 2DC score at weeks 12 and 24

*Protocol was revised to not include 25% improvement in the SRS-2 score as a measure of clinical response. 2DC, two domain composite; ABC, adaptive behavior composite; ADAMS, anxiety, depression, and mood scale; CGI-I, Clinical Global Impression Scale – Improvement; CGI-S, Clinical Global Impression Scale – Severity; HAM-A, Hamilton Anxiety Rating Scale; OACIS-I, Ohio Autism Clinical Impressions Scale – Improvement; OACIS-S, Ohio Autism Clinical Impressions Scale – Severity; PedsQL™, Pediatric Quality of Life Inventory; RBS-R, repetitive behaviors scale – revised; SRS-2, Social Responsiveness Scale, 2nd Edition; STAI, State-Trait Anxiety Inventory.
